# The role of salivary antioxidant level in polycystic ovary syndrome women under assisted reproductive technology treatment: A case-control study

**DOI:** 10.18502/ijrm.v22i12.18063

**Published:** 2025-01-31

**Authors:** Narges Gholizadeh, Maryam Koopaie, Ashraf Aleyasin, Atousa Mortazavi Milani, Marziyeh Aghahosseini, Mohammad Javad Kharrazifard, Mohadeseh Bahmaee

**Affiliations:** ^1^Department of Oral and Maxillofacial Medicine, School of Dentistry, Tehran University of Medical Sciences, Tehran, Iran.; ^2^Department of Infertility, Shariati Hospital, Tehran University of Medical Sciences, Tehran, Iran.; ^3^Clinical Research Development Unit of Imam Khomeini Hospital, Mazandaran University of Medical Sciences, Sari, Iran.; ^4^Department of Obstetrics and Gynecology and Female Infertility Unit, Tehran University of Medical Sciences, Tehran, Iran.; ^5^Department of Epidemiology and Biostatistics, Faculty of Public Health, Tehran University of Medical Sciences, Tehran, Iran.; ^6^School of Dentistry, Tehran University of Medical Sciences, Tehran, Iran.

**Keywords:** Infertility, Saliva, Antioxidant, Polycystic ovary syndrome, Diagnosis.

## Abstract

**Background:**

Infertility is defined as the inability to conceive after 12 months of regular unprotected intercourse. Approximately 85% of infertile couples have an identifiable cause, one of the most common causes of infertility is polycystic ovary syndrome (PCOS). The reduction of antioxidant levels in serum and follicular fluid in infertile women compared to healthy women shows the importance of further studying these markers.

**Objective:**

To study salivary and serum antioxidant levels in PCOS participants under assisted reproductive technology.

**Materials and Methods:**

This case-control study was conducted on 80 women in 2 groups including normal participants as control and PCOS groups (n = 40/each). Serum and salivary antioxidant levels such as saliva superoxide dismutase (SOD), saliva anti-Müllerian hormone (AMH), serum SOD, serum total oxidant status, and serum AMH were measured.

**Results:**

The average age of participants was 31.6 ± 5.4 yr. In both the saliva and serum, antioxidant levels differed significantly between the PCOS and control groups. Key findings showed that the PCOS group had different antioxidant levels and higher serum AMH levels compared to the control group, with all differences being statistically significant (p < 0.05).

**Conclusion:**

Our finding underscored that saliva antioxidant levels, especially SOD, are a good marker for PCOS diagnosis. It is noninvasive, can easily be performed by the participants, and can be collected in various settings without specialized equipment.

## 1. Introduction

Infertility is the inability to conceive after 12 months of regular, unprotected intercourse. About 85% of couples experiencing infertility have identifiable causes, such as ovulation disorders, male factor infertility, or fallopian tube issues. The remaining 15% have “unexplained infertility”. Ovulation disorders account for roughly 25% of infertility cases, with about 70% of women with anovulation diagnosed with polycystic ovary syndrome (PCOS) (1).

PCOS is the most common endocrine disorder affecting women in reproductive age and throughout their lifespan from adolescence to postmenopause. The prevalence of PCOS is estimated at 10–13%. The etiology of PCOS is much more complex (2, 3). Clinical manifestations are heterogeneous with reproductive, metabolic, and psychological characteristics, and women face delayed diagnosis and dissatisfaction with care internationally (4–6). The diagnosis of PCOS is based on specific criteria proposed by the Rotterdam Criteria or the Androgen Excess and PCOS Society criteria (7).

Oxidative stress (OS) is an imbalance between oxidants and antioxidants, resulting from excessive reactive oxygen species (ROS) that the body cannot adequately defend against. This can lead to DNA damage, cell apoptosis, and changes in gene expression and immune response (8). Importantly, OS plays a crucial role in the pathophysiology of a variety of gynecological disorders, including PCOS, endometriosis, unexplained infertility, and pre-eclampsia (9, 10). For infertility, OS plays a vital role in male and female infertility. In men, ROS can damage sperm DNA and impair sperm function, leading to reduced sperm quality and motility. In women, OS can affect oocyte quality and impair embryo implantation, which can lead to difficulty conceiving or maintaining a pregnancy (11). Studies suggest that OS may contribute to the development and progression of PCOS. Women with PCOS usually have higher levels of ROS and lower levels of antioxidants than the general population. This imbalance can lead to inflammation, insulin resistance, and hormonal disturbances, which are common features of PCOS (12). Therefore, OS, by playing a role in PCOS and infertility, affects reproductive performance, hormone levels, and overall health (13).

Infertility is a significant concern for today's societies, and understanding its contributing factors is essential. Assisted reproductive technology (ART) offers the highest pregnancy rates per cycle, highlighting the importance of recognizing the causes of infertility to improve treatment methods (14). Even with improvements in fertilization and implantation rates, ART cannot fully replicate natural pregnancy due to the lack of physiological defense mechanisms and external sources of ROS. Research shows that high levels of antioxidant capacity are crucial for successful conception, and insufficient levels may lead to poor-quality embryos (15).

Current studies on oxidants and antioxidants in infertility mainly focus on serum, follicular, and seminal fluids. However, examining these markers in saliva is essential due to its advantages, such as less invasiveness, high reproducibility, lower risk of infection transmission, and ease of collection and storage.

No studies have yet investigated oxidant and antioxidant levels in infertile women with PCOS. This study aims to explore the relationship between serum and salivary levels of these markers in PCOS participants undergoing ART.

## 2. Materials and Methods

This case-control study was conducted on 80 women divided into 2 groups based on ovarian reserve and infertility history, including the normal population as the control group (n = 40) and the PCOS group (n = 40) from July 2023 to March 2024 at the Omid Infertility Treatment Center affiliated with the Tehran University of Medical Sciences, Tehran, Iran.

The inclusion criteria were: aged between 18 and 40 yr, body mass index between 18 and 25 kg/m^2^, confirmed diagnosis of PCOS based on Rotterdam criteria, and absence of systemic and chronic disease (7). Participants with a history of chemotherapy, abdominopelvic radiotherapy, autoimmune and rheumatological diseases, anti-inflammatory drugs consumed, endometriosis, and uterine myoma surgery, were excluded from the study. As with the PCOS participants, a normal population with a similar age and body mass index was included in the study with a 1:1 ratio.

Initially, the women qualified for this treatment were confirmed by 2 gynecologists, and then they were interviewed face-to-face by the conductors of the research. The study procedures were explained to them, in case of consent to enter the study and ensure appropriate compliance for follow-up and study procedures, they were grouped for conducting the research. Participants were divided into 2 groups based on ovarian reserve and infertility history: normal population (with normal ovarian function and no history of infertility) and PCOS (based on Rotterdam criteria and at least 12 months of infertility history) (7). The treatment protocol was set as an antagonist protocol for PCOS participants, who were referred to our infertility center for ART treatment (16).

Saliva and serum antioxidant levels were measured when the luteinizing hormone surge triggered (gonadotropin-releasing hormone agonist injection day), and the relationship between the measured antioxidant levels was checked. The samples were prepared on the day of human chorionic gonadotropin injection for puncture to check the levels of antioxidants.

The saliva sample was collected using the spitting method. The participants were asked to avoid eating and drinking for 90 min before taking the sample and then collect saliva in 60 sec intervals for 5–15 min and pour it into pre-weighed sterile containers. Saliva samples were centrifuged at a speed of 2500 g and kept in a freezer at minus 80 C until the time of examination by the enzyme-linked immunosorbent assay. For the serum sample, 5 ml of peripheral blood was taken from the participants through regular syringes and collected in a test tube.

### Ethical Considerations

The ethics committee of Tehran University of Medical Sciences, Tehran, Iran approved this study (Code: IR.TUMS.DENTISTRY.REC.1402.041). During the research period, all eligible participants were included in the study after obtaining the written informed consent.

### Statistical Analysis

Data analysis was conducted in 2 steps: descriptive and inferential statistics, employing a comprehensive approach to elucidate the study's findings.

#### Descriptive statistics

In the descriptive analysis phase, the data were thoroughly examined to provide a foundational understanding of the variables under study. For qualitative data, measures such as frequency and percentage were calculated. Quantitative data were analyzed using central tendency and variability measures, including, mean, median, variance, and standard deviation, to illustrate the distribution and spread of numerical data. This phase aimed to summarize and present the data in a manner that highlights underlying patterns and characteristics without making inferential conclusions.

#### Inferential statistics

The inferential statistics segment focused on drawing conclusions and making predictions about the population based on the sample data. This phase involved the application of statistical tests to examine the hypotheses and assess relationships between variables. Parametric tests, such as the one-sample *t* test, independent *t* test, paired *t* test, and ANOVA, were used when the data met specific assumptions, including normality and homogeneity of variance. For datasets not meeting these criteria, nonparametric alternatives like the Mann-Whitney U test, Chi-square test, Wilcoxon signed-rank test, and Kruskal-Wallis H test were employed. These tests were carefully chosen based on the nature of the data and the specific research questions, ensuring the integrity and validity of the statistical inferences. Finally, the ROC curve was used to figure out the sensitivity and specificity of the results of each marker.

All analyses were conducted using Statistical Package for the Social Sciences, version 27.0, SPSS Inc., Chicago, Illinois, USA (SPSS). A significance level of 0.05 was adopted for all tests, meaning that results achieving a p-value less than this threshold were considered statistically significant. This significance level was chosen to balance the risks of type I and type II errors, aiming to minimize the likelihood of falsely declaring a finding as significant or overlooking a true effect.

## 3. Results

The study included 80 participants in 2 groups of 40, PCOS and the control group (normal participants). The average age of participants was 31.60 ± 5.362 yr, ranging from 18–40 yr old. Also, the average infertility time of the PCOS participants was 2.78 ± 2.862 yr, ranging from 1–12 yr. Other demographic characteristics, such as the number of metaphases, 2 oocytes, and fetus with a breakdown of participant groups, are available in table I.

The enzyme-linked immunosorbent assay test determined saliva and serum antioxidant levels. Statistical analysis revealed significant differences between groups in saliva SOD, serum SOD, serum TOS, and serum anti-Müllerian hormone (AMH) (Table II).

Also, PCOS participants showed lower levels in saliva SOD but upper levels in serum SOD, serum TOS, and serum AMH (Table II).

Statistical analysis shows that saliva SOD shows a positive correlation with serum total antioxidant capacity and a negative correlation with serum total oxidant status and serum AMH with statistically significant levels (Table III).

**Figure 1:**
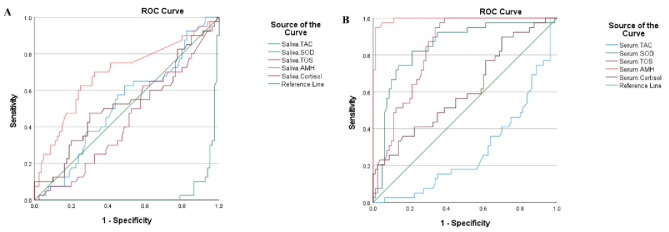
Specificity and sensitivity of A) Saliva and B) Serum antioxidant level. TAC: Total antioxidant capacity, SOD: Superoxide dismutase, TOS: Total oxidant status, AMH: Anti-Müllerian hormone, ROC curve: Receiver operating characteristic curve.

Also, ROC curve analysis between PCOs groups shows that saliva SOD and serum AMH are good markers with significant specificity and sensitivity, so they may be suitable markers for PCOS participant's diagnosis (Figure 1).

**Table 1:** Demographic characteristics of participants

**Group**	**Normal**	**PCOS**	**Total**	**P-value**
**Age (yr)**	32.55 ± 5.439 (21–40)	30.65 ± 5.177 (18–40)	31.60 ± 5.362 (18–40)	0.114 (-1.600)
**Infertility duration (yr)**	-	2.78 ± 2.862 (1–12)	2.78 ± 2.862 (1–12)	-
**Metaphase 2 oocyte (number)**	-	17.02 ± 11.761 (0–65)	17.02 ± 11.761 (0–65)	-
**Fetus (number)**	-	14.33 ± 9.056 (0–46)	14.33 ± 9.056 (0–46)	-
Data presented as Mean ± SD (min-max). Chi-square test, PCOS: Polycystic ovary syndrome				

**Table 2:** Comparison of serum and salivary antioxidant levels between participants

**Variables**		**Groups**		**F**	**P-value**
		**PCOS**	**Normal**		
**Saliva**					
	**TAC**	0.23 ± 0.173 (0.17–0.28)	0.22 ± 0.180 (0.16–0.27)	0.084	0.772
	**SOD**	50.88 ± 4.333 (49.50–52.27)	59.47 ± 3.993 (58.19–60.74)	84.932	< 0.001
	**TOS**	9.29 ± 12.102 (5.41–13.16)	10.86 ± 14.725 (6.15–15.57)	0.272	0.603
	**AMH**	0.60 ± 1.630 (0.08–1.12)	0.11 ± 0.150 (0.06–0.16)	3.499	0.065
	**Cortisol**	7.69 ± 3.972 (6.42–8.96)	6.54 ± 2.302 (5.80–7.27)	2.528	0.116
**Serum**					
	**TAC**	0.28 ± 0.073 (0.25–0.30)	0.33 ± 0.077 (0.30–0.35)	8.496	0.005
	**SOD**	54.02 ± 7.977 (51.44–56.61)	47.23 ± 6.559 (45.13–49.32)	17.157	< 0.001
	**TOS**	61.66 ± 9.418 (58.65–64.67)	50.46 ± 19.728 (44.15–56.77)	10.502	0.002
	**AMH**	8.19 ± 3.281 (7.14–9.24)	2.77 ± 1.030 (2.44–3.09)	99.524	< 0.001
	**Cortisol**	296.32 ± 141.638 (251.02–341.62)	242.39 ± 108.457 (207.71–277.08)	3.655	0.06
Data presented as Mean ± SD (95% CI). One-way ANOVA. PCOS: Polycystic ovary syndrome, TAC: Total antioxidant capacity, SOD: Superoxide dismutase, TOS: Total oxidant status, AMH: Anti-Müllerian hormone					

**Table 3:** Correlation between serum and salivary antioxidant level

**Variables**			**Salvia**					**Serum**				
			**TAC**	**SOD**	**TOS**	**AMH**	**Cortisol**	**TAC**	**SOD**	**TOS**	**AMH**	**Cortisol**
**Saliva**												
	**TAC**	**Pearson correlation**	1	0.07	-0.12	0.01	0.04	0.05	-0.06	0.11	-0.05	0.23
		**P-value**	-	0.558	0.29	0.92	0.70	0.64	0.58	0.35	0.67	0.84
	**SOD**	**Pearson correlation**	0.66	1	0.16	-0.20	-.08	0.23	-0.21	-0.40	-0.52	-0.10
		**P-value**	0.56	-	0.16	0.08	0.47	0.04	0.07	< 0.01	< 0.01	0.38
**Saliva**												
	**TOS**	**Pearson correlation**	-0.12	0.16	1	-0.09	-0.14	-0.03	-0.01	-0.01	-0.11	-0.04
		**P-value**	0.25	0.16	-	0.43	0.22	0.82	0.91	0.96	0.35	0.97
	**AMH**	**Pearson correlation**	0.01	-0.20	-0.09	1	0.04	0.03	0.07	0.10	0.20	0.22
		**P-value**	0.92	0.08	0.41	-	0.72	0.83	0.57	0.40	0.08	0.05
	**Cortisol**	**Pearson correlation**	0.04	-0.08	-0.14	0.04	1	0.14	0.25	-0.07	0.13	0.60
		**P-value**	0.70	0.47	0.22	0.72	-	0.22	0.04	0.53	0.27	< 0.01
**Serum**												
	**TAC**	**Pearson correlation**	0.05	0.23	-0.03	0.03	0.14	1	-0.08	-0.15	-0.14	0.23
		**P-value**	0.64	0.04	0.82	0.83	0.22	-	0.51	0.19	0.22	0.04
	**SOD**	**Pearson correlation**	-0.06	-0.21	-0.01	0.07	0.25	-0.08	1	0.10	0.37	0.36
		**P-value**	0.58	0.07	0.91	0.57	0.02	0.51	-	0.37	< 0.01	< 0.01
	**TOS**	**Pearson correlation**	0.11	-0.40	-0.01	0.10	-0.07	0.15	0.10	1	0.26	0.02
		**P-value**	0.35	< 0.01	-0.96	0.40	0.53	0.19	0.37	-	0.02	0.89
	**AMH**	**Pearson correlation**	-0.05	-0.52	-0.11	0.20	0.13	-0.14	0.37	0.26	1	0.23
		**P-value**	0.67	< 0.01	0.35	0.08	0.27	0.22	< 0.01	0.02	-	0.04
	**Cortisol**	**Pearson correlation**	0.02	-0.10	0.004	0.22	0.60	0.23	0.36	0.02	0.23	1
		**P-value**	0.84	0.38	0.97	0.052	< 0.01	0.04	< 0.01	0.89	0.04	-
Pearson correlation coefficient test. TAC: Total antioxidant capacity, SOD: Superoxide dismutase, TOS: Total oxidant status, AMH: Anti-Müllerian hormone												

## 4. Discussion

Our research revealed significant differences in antioxidant levels between the PCOS group and the control group, both in saliva and serum samples. Specifically, individuals in the PCOS group displayed altered antioxidant concentrations and had elevated serum levels of AMH when compared to the control group. Importantly, most of the observed differences were statistically significant, with a p-value of less than 0.05, highlighting the relevance of these findings in understanding the biochemical variations associated with PCOS.

As mentioned, OS has been identified and introduced as a potential contributing factor in the development and progression of PCOS, as women with PCOS have higher levels of ROS and lower levels of antioxidants compared to the normal population (12–15). This study is among the first to examine salivary antioxidants in PCOS participants undergoing ART. Women with PCOS have elevated levels of AMH, a glycoprotein secreted by ovarian follicle cells, correlating strongly with ultrasound-detected follicle counts. The findings indicate that serum AMH is significantly related to PCOS incidence, with a sensitivity of 95% and specificity of 92% for diagnosing PCOS in in vitro fertilization (IVF) participants. Recent systematic reviews confirm that serum AMH levels are reliable markers for PCOS diagnosis (17). Other studies have shown that increased AMH serum levels in PCOS participants are associated with a lower probability of response to IVF treatment (18). These results are consistent with our study results. However, no study has been done on the salivary levels of AMH; the present study showed the difference in salivary AMH levels between the groups, even considering that this level did not reach a significant level, it may be a suitable indicator in PCOS participants, which should be confirmed with more extensive studies in the future.

SOD is an antioxidant enzyme that protects against infertility. This study found that serum SOD levels are higher in women with PCOS compared to those without, while salivary SOD levels are lower in the PCOS group. Elevated serum SOD may indicate a protective response to oxidative damage in PCOS.

Our findings suggest that salivary SOD is a more effective marker than serum SOD for predicting responses to infertility treatment and diagnosing PCOS, as it demonstrates greater sensitivity and specificity. This study is the first to compare salivary and serum SOD levels, supporting the idea that salivary SOD is the better diagnostic tool. In contrast, earlier research indicated decreased serum SOD levels in women with PCOS, which is inconsistent with our results (19). But Talat et al. showed that serum SOD levels were higher in women with PCOS compared to the control group. This study was a meta-analysis study, the results of which align with our research (20). In addition, SOD is related to hormonal changes after menopause, so salivary SOD levels in postmenopausal women with periodontitis are lower than in nonmenopausal women without periodontitis, which confirms the protective role of saliva.

Among the other results of this study, it can be mentioned that among the markers, salivary SOD has a significant positive relationship with the number of infertilities, which means that with the increase in the number of years of infertility, SOD levels also increase. Based on the study results of Pekel et al. SOD activity in unexplained infertility, PCOS, and endometriosis groups was significantly higher than the control group, which was consistent with the present study (21). The relationship between SOD and infertility is rooted in OS and its implications for reproductive health. SOD is an important antioxidant enzyme that catalyzes the conversion of superoxide radicals to oxygen and hydrogen peroxide, thereby reducing OS in cells. Infertility can be affected by OS, which occurs due to an imbalance between the formation of ROS and the body's ability to detoxify them. High levels of ROS can damage sperm, oocytes, and reproductive tissues and affect fertility (22).

Fewer studies have focused on salivary OS markers; but several have investigated psychological stress markers, like cortisol and sex hormones (estrogen and progesterone), in the saliva of ART participants. Salivary cortisol accurately reflects serum-free cortisol, the biologically active form of the hormone, making it a more precise measure than total serum cortisol. However, the research shows significant variability due to differences in collection times, treatment stages, and methods for saliva handling and diagnosis. These inconsistencies can affect results, leading to the conclusion that cortisol is not a suitable marker for this study (23).

Age is one of the crucial factors in fertility and affects the levels of oxidant and antioxidant markers. This relationship can directly affect the quality and ability of fertility. Some aspects of this relationship are discussed below: with age, the level of oxidants in the body also increases, especially in the reproductive parts. These oxidants, which include free radicals, can damage egg and sperm cells. Oxidative damage can lead to a decrease in the quality of eggs and sperm, a decrease in fertility, and an increase in the risk of miscarriage. Antioxidant patterns in women at the age of 37 change toward an increase in the level of oxidants and a decrease in the level of antioxidants, which can disrupt the efficiency of ROS clearance (24).

Considering the wide range of systemic factors affecting OS, the use of specific markers that have a local secretion source in saliva can be more specific and sensitive in predicting the results of ART. For participants undergoing ART, salivary diagnostic tests are attractive alternatives with less stress, noninvasive, and with less intervention. Saliva samples can be stored for a week at room temperature and for a longer period in the freezer. Salivary markers in the diagnosis of infertility offer several potential advantages that make them an attractive tool for participants and healthcare providers. Saliva collection is noninvasive and easily performed by the participants. Saliva samples can be collected in a variety of settings -at home, at work, or in a clinical setting- without the need for specialized equipment. Saliva can be used to measure the level of hormones such as progesterone, estrogen, and testosterone. Monitoring these hormones can provide insight into ovulation, menstrual cycle regularity, and overall reproductive health. Because saliva collection is noninvasive, it has a lower risk of infection than methods that involve needles or other invasive techniques (25). Different levels of OS markers are necessary for the success of various stages of IVF. For example, during embryo transfer, higher oxidant levels and lower antioxidant levels are needed, and saliva may be a more accurate medium than serum for detecting these changes. This study focuses on how serum and salivary ROS markers can predict clinical pregnancy. Successful IVF involves multiple stages, including folliculogenesis, oocyte maturation, fertilization, and embryo implantation. To better understand the role of ROS in ART, it is more effective to consider clinical pregnancy as the main outcome rather than just metrics like egg development or fertilization rates (26).

This study is one of the first to present encouraging data on salivary antioxidant levels and their potential diagnostic uses, which should be validated in future research. We recommend that further studies examine the impact of these levels on ART outcomes in PCOS, poor responders, and normal responders, along with embryo quality, clinical pregnancy rates, and neonatal outcomes. A key limitation of this study is its single-center design and small sample size.

## 5. Conclusion

Following recent studies, our finding underscores that saliva antioxidant levels, especially superoxide dismutase, are a good marker for PCOS diagnosis. It is noninvasive and easily performed by the participants and can be collected in various settings without specialized equipment.

##  Data Availability

Data will be made available for review or query upon reasonable request from the corresponding author.

##  Author Contributions

A. Aleyasin, M. Bahmaee, N. Gholizadeh, and M. Koopaie: Conceptualization, methodology, writing-review, and editing. A. Mortazavi Milani: Conceptualization, methodology, formal analysis, investigation, writing-original draft preparation-review and editing. MJ. Kharrazifard: Formal analysis. M. Aghahosseini: Methodology and investigation. Critical revision of the manuscript for important intellectual content: All authors.

##  Conflict of Interest

The authors declare that there is no conflict of interest.
